# Bibliometric and visual analyses of advancements in chronic kidney disease management

**DOI:** 10.1097/MD.0000000000038576

**Published:** 2024-06-28

**Authors:** Li Dong, Lian Tan

**Affiliations:** a Department of Nephrology, Yongchuan Hospital of Chongqing Medical University, Chongqing, China; b Department of Stomatology, The Second Affiliated Hospital of Chongqing Medical University, Chongqing, China.

**Keywords:** bibliometrics, chronic disease management, chronic kidney disease, visual analysis

## Abstract

Chronic kidney disease (CKD) is characterized by high incidence, prolonged course, significant health damage, and a heavy societal burden. Understanding the history and content of CKD research is crucial to further its recognition and management, in addition to reducing its individual and societal burdens. This study aimed to assess the management history of CKD to provide a foundation for clinical medical staff to systematically understand its evolution. The Web of Science Core Collection database was screened for CKD management studies published between January 1, 1948, and December 31, 2021. From the search results, we performed statistical descriptions of the publication date, volume, and type. Using VOS-viewer 1.6.19, variables from the included articles were obtained for keyword co-occurrence clustering and sequence analyses to determine research themes, segment phases based on publication volumes over varied timeframes, assess the dynamic progression of CKD management, and anticipate future research trends. In total, 26,133 articles met the inclusion criteria. The analysis revealed 3 stages of CKD management research: the slow development stage (1948–1998), which was initiated by epidemiological studies without ideal clustering; the steady growth stage (1999–2010), which was focused on CKD complication management and quality-of-life research; and the rapid development stage (2011–2022), which was dominated by 7 major clusters, mainly regarding the treatment and management of severe conditions and management patterns. The CKD research journey is comprised of 3 stages, the contents of which form an interconnected research model. Future research should focus on the establishment of management models and the application of intelligent management tools. Furthermore, this work can serve as a reference for the further expansion of research in this field and in improving its management.

## 1. Introduction

Chronic kidney disease (CKD) is characterized by chronic renal structural and functional dysfunction; a history of renal damage (>3 months) due to a variety of causes, including pathological damage; a normal or abnormal glomerular filtration rate; abnormalities in the blood or urine composition; imaging abnormalities; or an unexplained decline in the glomerular filtration rate (<60 [mL/min]/1.73 m^2^) for >3 months.^[1]^ CKD is prevalent worldwide, and its incidence has been increasing every year with the growing aging population and increasing rates of hypertension and diabetes.^[2]^ During the end stages of CKD, hospitalization and mortality rates remain high, even with the application of peritoneal dialysis, hemodialysis, and other renal replacement therapies.^[3,4]^ This is particularly true in developing countries, where the survival rates and lifespans of patients with end-stage renal disease are alarmingly low.^[5]^ Long-term sustained treatment and repeated hospitalizations impose significant burdens on both the patients and the community. Subsequently, CKD has become a major global health concern.^[6,7]^

In 2002, the National Kidney Foundation-Kidney Disease Outcomes Quality Initiative introduced a conceptual framework for CKD management using established clinical practice guidelines to assess, stage, and stratify CKD.^[8]^ Building on this foundation, in 2012, the International Organization for Kidney Disease: Improving Global Outcomes issued guidelines that refined, revised, and updated the stages, progression, evaluations based on stages, and management of CKD. These guidelines also emphasize the importance of preventing CKD etiological factors and lifestyle changes in patients with CKD.^[9]^ In 2021 and 2022, the Kidney Disease: Improving Global Outcomes introduced management practice guidelines for glomerular diseases and diabetic nephropathy. Therefore, the approach to CKD management has gradually transitioned from broader control to more refined management tailored to specific diseases.^[10,11]^ Understanding the current status and principles of CKD management is instrumental in gaining a deeper understanding of CKD, strengthening its management capabilities, enhancing the quality of life of patients, and effectively controlling medical costs.

Using publications over the course of different time-periods as the basis for phase division, this study aimed to examine the global development and research hotspots of CKD management. The study outcomes will help to predict future research trends in CKD management and provide a reference for subsequent research directions.

## 2. Methods

### 2.1. Literature retrieval and selection

The Medical Subject Headings were referred to for subject terms and free words related to the management of CKD. Subject term searches were performed in the Web of Science core and Medline databases using Boolean logic.

Using the advanced search mode, the following combinations of topic searches (TS) were used: TS = (chronic kidney disease) OR TS = (chronic renal disease) OR TS = (chronic renal failure) AND TS = (disease management) OR TS = (chronic disease management).

This retrospective study used data obtained for clinical purposes. The Ethics Committee of Yongchuan Hospital, Chongqing Medical University waived the need for ethical approval.

### 2.2. Inclusion and exclusion criteria

The study inclusion criteria were literature related to the management of CKD; the timeframe for the search was from the creation of the database to December 31, 2022; the language of included studies was limited to English; and there was no restriction on the type of article; automatic detection of duplicates was done using EndNote X9. The articles were screened independently by 2 researchers through quick reading of the titles and abstracts. In the case of a disagreement, it was resolved by negotiation.

The exclusion criteria were duplicate publications, including conference summaries and full papers published in the same study; and unpublished studies.

### 2.3. Bibliometric analysis and visualization

The “Analyze Search Results” feature of the Web of Science search platform was used to collect information from the literature. Data were categorized according to publication year and document type. Descriptive statistics were computed, and the data were exported and processed using Microsoft Excel 2016 (Research Resource Identifier: SCR_016137). The documents were then exported in “.txt” format and Endnote X9 was used to automatically detect duplicate articles. After filtering, the data were input into VOSviewer 1.6.19 (Research Resource Identifier: SCR_023516) for visualization. Keywords and related information were extracted and reviewed. First, similar keywords were consolidated, and incorrect or misleading keywords were removed. The keywords were then subjected to co-occurrence clustering and time series analyses. To understand the evolving themes of CKD management research across different periods, the studies were categorized into 3 stages according to literature inclusion and the publishing trends of the database. The advantages and primary areas of CKD research were explored by examining publication counts, document types, keyword co-occurrence clustering, and time-series analyses.

## 3. Results

### 3.1. Bibliometric overview of the research themes

**Figure 1.**
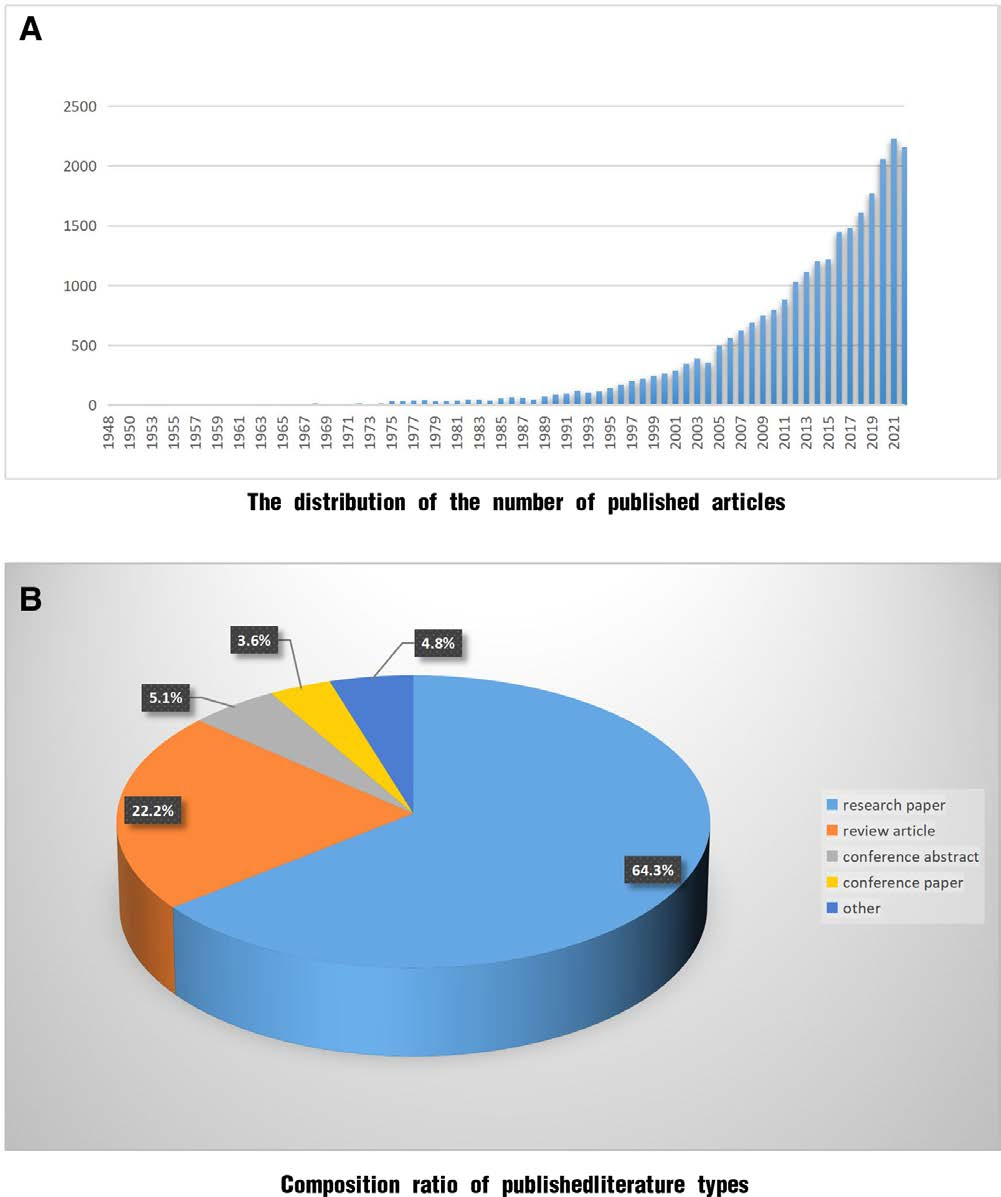
Bibliometric overview. (A) The distribution of the number of published articles; (B) composition ratio of published literature types.

In this study, 26,133 articles that met the inclusion criteria were selected from the Web of Science search platform. The earliest articles were published in 1948 (n = 2). Over time, the number of publications gradually increased (Fig. 1A). Until 1991, the annual publication rate of CKD management-related articles remained relatively low (<100 articles/year); by 1998, the number of articles had increased to 223 articles/year. There was no core database for articles published between 1948 and 1998; therefore, searches were primarily conducted using the Medline database. Between 1999 and 2010, there was an upward trend in the publication rate, ranging from 224 to 797 articles/year. From 2011 to 2022, publication growth increased considerably, averaging approximately 1000 articles/year, with a peak of 2231 articles in 2021. Accordingly, CKD management research was divided into the following 3 phases: slow development (1948–1998), steady growth (1999–2010), and rapid development (2011–2022). Of the 26,133 published articles, 16,804 were research articles (64.3%) and 5799 were review-type articles (22.2%), with conference abstracts, conference papers, and other types of articles accounting for 5.1%, 3.6%, and 4.8%, respectively (Fig. 1B).

### 3.2. Slow development phase (1948–1998)

#### 3.2.1. Trends in the volume of the literature

**Figure 2.**
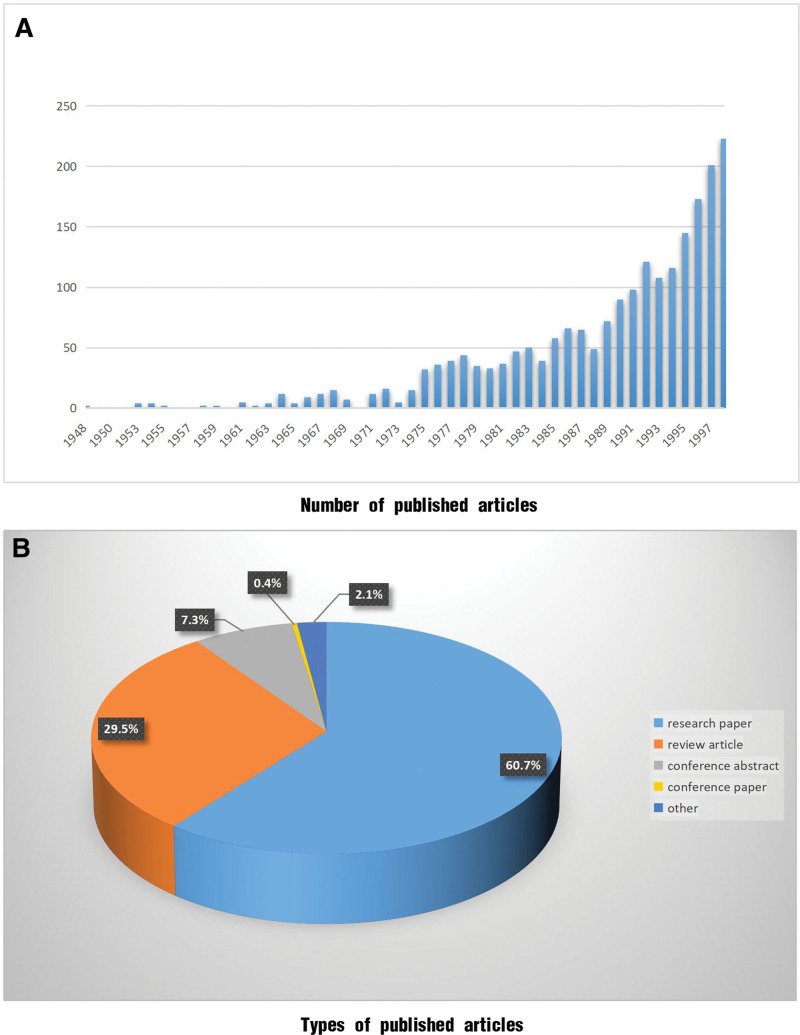
Publication status between 1948 and 1998. (A) Number of published articles; (B) types of published articles.

Between 1948 and 1998, 2118 research articles were published; the specific details are provided in Figure 2A. Growth in the number of published articles during this phase was slow, especially before 1973, when the annual number of publications was <10. However, as the attention to CKD increased, the number of published articles approached 100 by 1991. During this phase, primary research dominated the types of published articles, followed by review-type articles (Fig. 2B).

#### 3.2.2. Keyword clustering and temporal analyses

**Figure 3.**
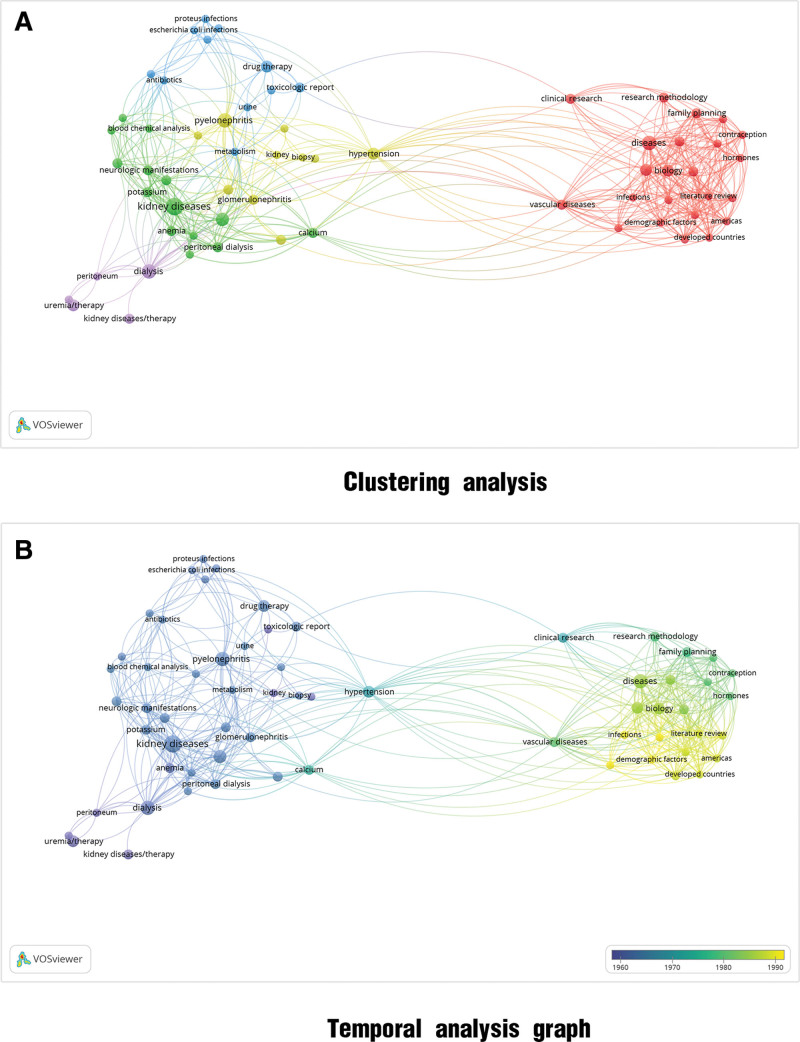
Keyword cluster analysis of articles published between 1948 and 1998. (A) Clustering analysis. (B) Temporal analysis graph.

A clustering analysis based on the research keywords is shown in Figure 3A. Owing to the limited number of articles published during this phase, a well-defined cluster was not formed. As shown in the temporal graph (Fig. 3B), few early CKD management-related research articles were available, and no corresponding system emerged. No corresponding nodes were observed in the temporal graph. The time-adjustment node was set from 1960 to 1990. During the 1990s, research focused mainly on management in developed countries, and the focus on demographic factors and other epidemiological investigations increased.

### 3.3. Steady growth phase (1999–2010)

#### 3.3.1. Trends in the volume of the literature

**Figure 4.**
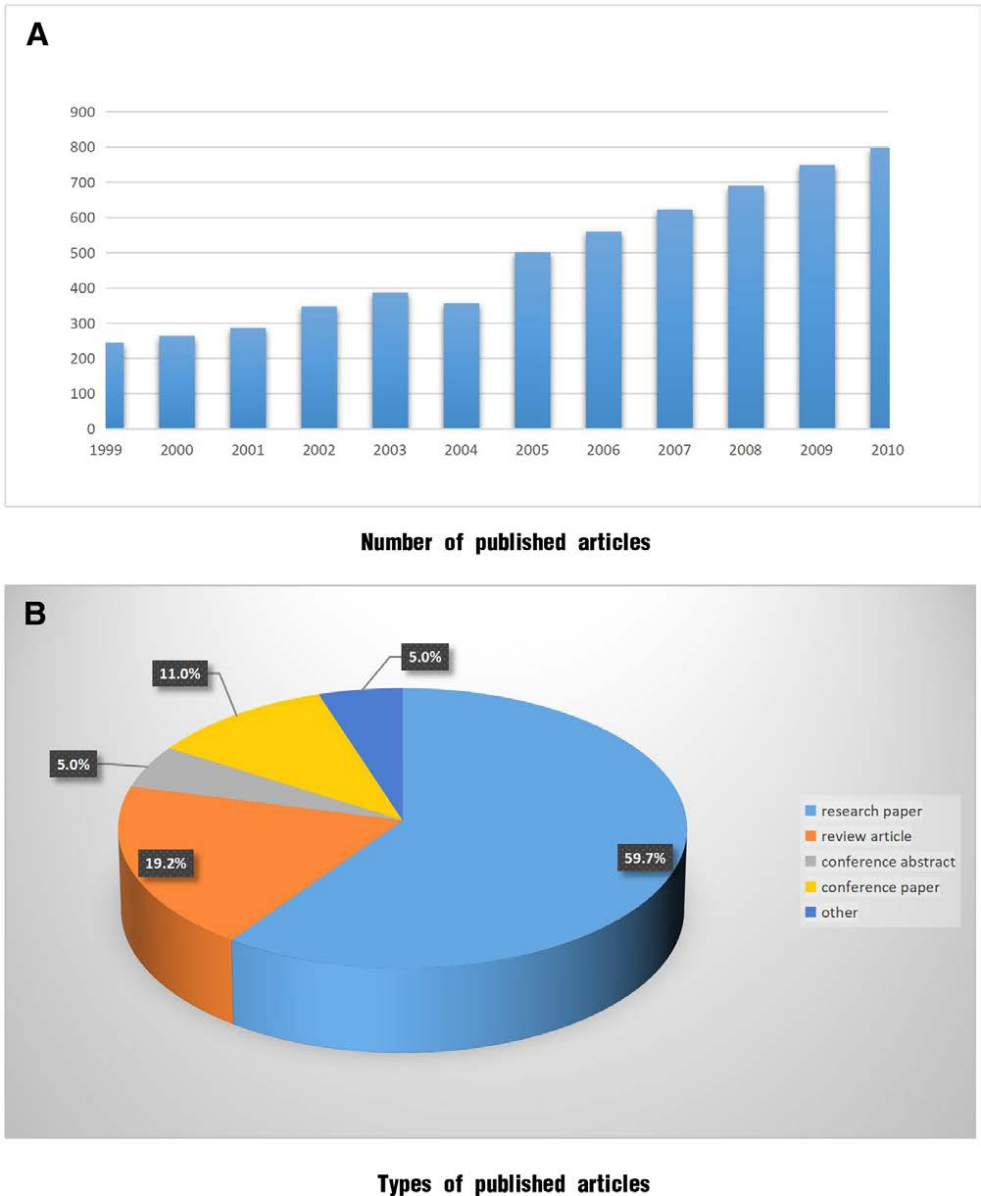
Publication status between 1999 and 2010. (A) Number of published articles; (B) types of published articles.

Between 1999 and 2010, 5807 research articles were published. The annual publication details are shown in Figure 4A. From 1999 to 2003, the number of publications remained stable at <400 articles/year; however, in 2004, the publication rate grew to >500 articles/year and remained relatively high for the subsequent 5 years (560–797 articles/year). During this period, the published articles primarily consisted of research articles (59.7%), followed by review articles (19.2%), conference papers (11%), and conference abstracts (5%) (Fig. 4B).

#### 3.3.2. Keyword clustering and temporal analyses

**Figure 5.**
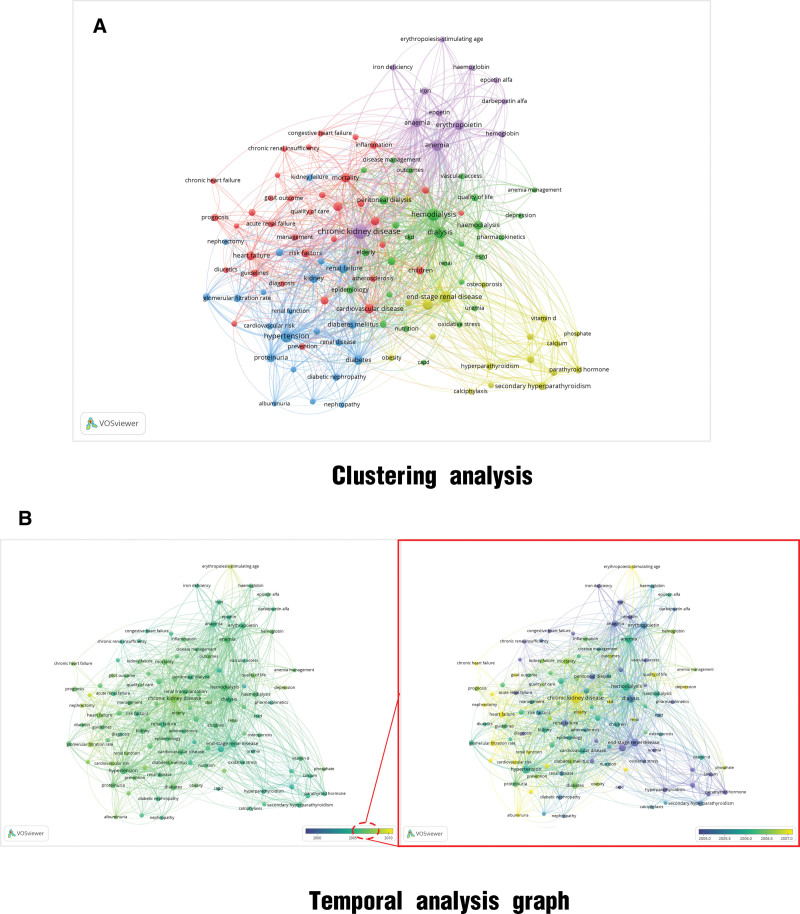
Keyword cluster analysis of articles published between 1991 and 2000. (A) Clustering analysis. (B) Temporal analysis graph.

A clustering diagram is shown in Figure 5A. During this phase, the research was divided into the following 5 clusters: Cluster 1 (purple) was related to the management of anemia-related complications; Cluster 2 (red) focused on cardiovascular disease management; Cluster 3 (blue) focused on diabetic nephropathy management; Cluster 4 (yellow) was related to the regulation of calcium and phosphorus metabolism; and Cluster 5 (green) pertained to dialysis and quality of life. As shown in the temporal graph (Fig. 5B), the research hotspots were concentrated between 2005 and 2007. Before 2005, progress in CKD management research was relatively slow. Adjusting the time setting to allow a closer examination of the temporal graph revealed that researchers began to focus on the management of CKD complications, such as cardiovascular diseases. Furthermore, case screening and quality of life have emerged as new research topics.

### 3.4. Rapid development phase (2011–2022)

#### 3.4.1. Trends in the volume of the literature

**Figure 6.**
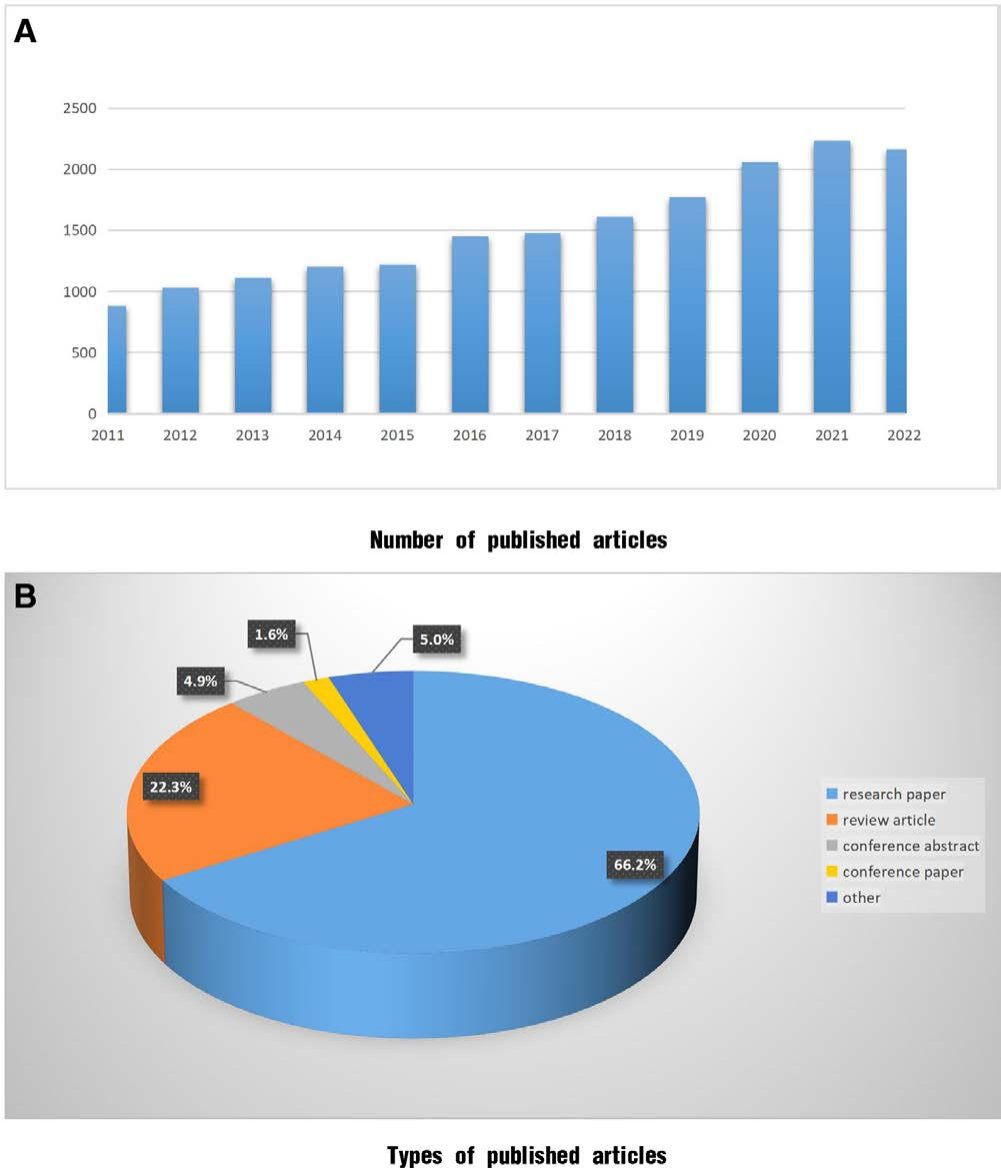
Publication status between 2011 and 2022. (A) Number of published articles; (B)Types of published articles.

Between 2011 and 2022, 18,208 articles were published, marking a period with explosive growth. In 2011, the publication rate consistently exceeded 1000 articles/year. This number surpassed 2000 articles in 2020 and reached a historic peak of 2231 articles in 2021 (Fig. 6A). Research on CKD management had garnered widespread academic attention. During this period, the articles primarily consisted of original research articles (66.2%), whereas the proportion of review articles increased from the previous phases to 22.3%. Conference articles and abstracts were the next most common article types. A breakdown of the article types is depicted in Figure 6B.

#### 3.4.2. Keyword clustering and temporal analyses

**Figure 7.**
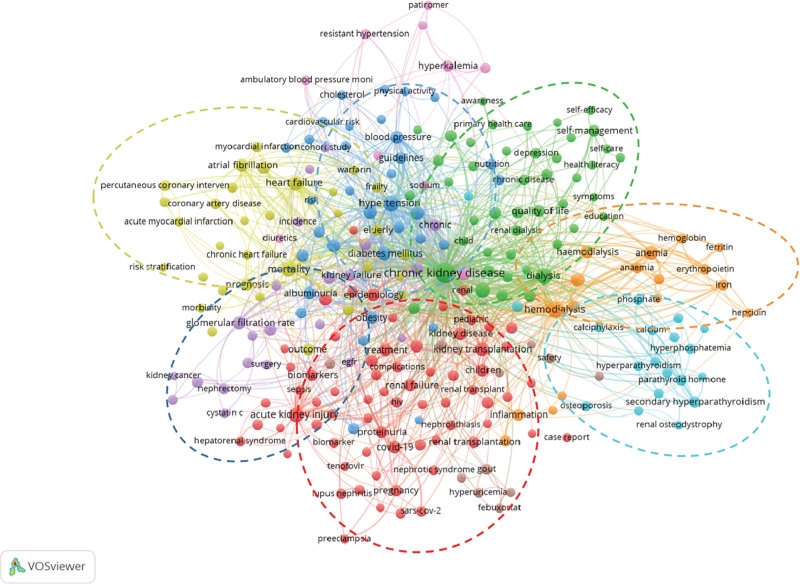
Clustering analysis of articles published between 2011 and 2022.

A clustering diagram of literature published between 2011 and 2022 is shown in Figure 7. Seven major clusters were identified in this study. Cluster red (treatment and management of critical care), including the management and treatment of severe conditions such as hepatorenal syndrome and acute kidney injury. Notably, coronavirus disease 2019 (COVID-19) appeared in the cluster analysis during this period. Cluster purple (surgical treatment), including partial nephrectomy, surgery for renal cancer, and nephrectomy. Cluster (yellow) (Management of Complicated Cardiovascular Diseases), which included management of cardiovascular disease complications, such as heart failure, acute coronary syndrome, and coronary artery disease, and studies focused on anticoagulant treatments, mortality rates, and predictions. Cluster dark blue (disease guidelines and risk management), including those focused on hypertension, diabetes, and related risk factors and assessments. Cluster green (Health Management Model), which included health management methods that primarily focused on self-management, health relationship management, telemedicine, and primary care. Factors that influence self-management, such as disease awareness, educational level, and patient quality of life, were also investigated. Cluster orange (relationship between different dialysis modalities and anemia), including hemodialysis and peritoneal dialysis. Finally, cluster light blue (management of calcium and phosphorus metabolic homeostasis), including conditions such as hypoparathyroidism, hyperparathyroidism, vascular calcification, and abnormal bone density. The magenta and brown zones of the diagram contain fewer studies and did not form well-defined cluster systems.

**Figure 8.**
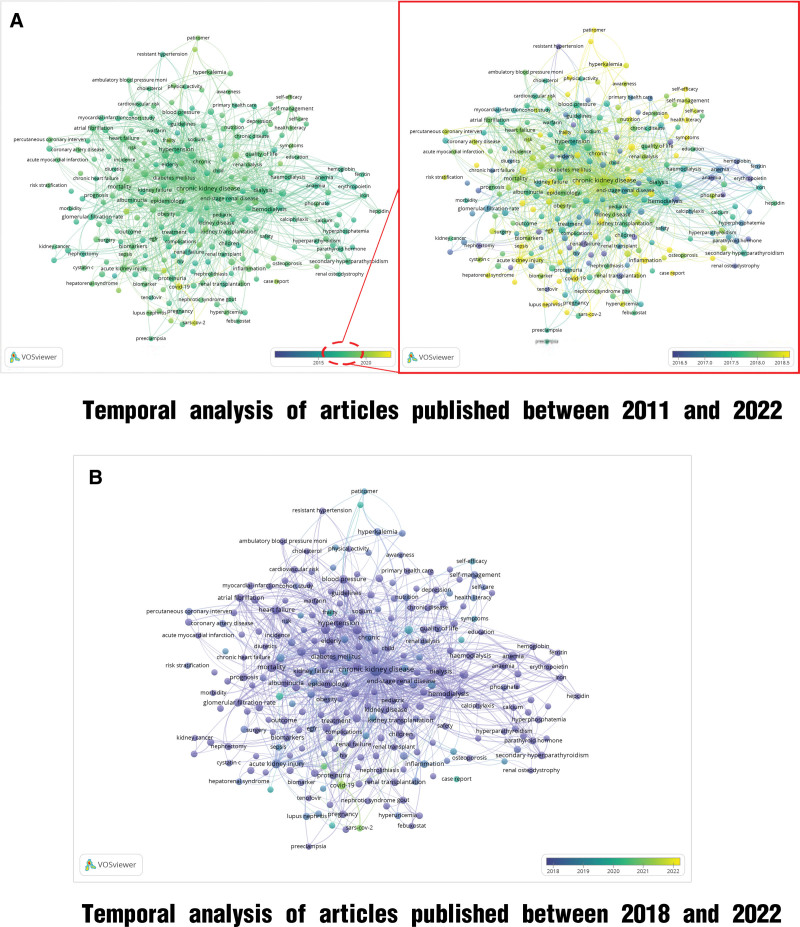
Temporal analysis between 2011 and 2022. (A) Temporal analysis of articles published between 2011 and 2022. (B) Temporal analysis of articles published between 2018 and 2022.

A temporal analysis based on the keywords from studies published between 2011 and 2022 is shown in Figure 8A. The primary occurrence of the keywords during this phase was observed between 2016 and 2018. After narrowing the timeframe for a closer analysis, the research hotspots included patient health management models, severe disease management, chronic disease case management, and telemedicine. Adjustment of the timeline to review the most recent 4 years (2018–2022) revealed that after 2019, the emphasis of research primarily shifted toward renal complications arising from COVID-19 (Fig. 8B).

## 4. Discussion

We conducted a retrospective literature analysis on CKD management based on keyword clustering and topic mining. CKD management plays a particularly important role in slowing disease progression, and studies on CKD management have gradually attracted the attention of many researchers in the field.^[12,13]^ From the perspective of the publication types, in the early stage, the management of CKD was in the primitive research period, consisting mainly of original papers. With the continuous increase in the depth of research and emergence of relevant literature, the proportion of review articles and conference papers has gradually increased. Therefore, after decades of development, the management of CKD has gradually matured and formed a system. Research related to CKD management rapidly developed after 2011, especially between 2018 and 2022, with >1500 papers published annually. This result corresponds to the rapid global increase in CKD incidence rates,^[14]^ which had reached 10% by 2020. In Asia, the estimated adult population of patients with CKD was as high as 434.3 million, with China (159.8 million) and India (140.2 million) accounting for 69.1% of the total number of patients with CKD in this region.^[15]^ Research on CKD management has gradually spread from developed countries to developing ones. In recent decades, researchers have increased the depth of their understanding and study of disease management, and the focal points and models of CKD management have also evolved.

This study was divided into 3 phases based on the publication volume of CKD management-related research. Phase one was a slow development stage (1948–1998). The earliest study was published in 1948 in the *Wisconsin Medical Journal*.^[16]^ This article initiated a study of CKD management through research on the management of chronic glomerulonephritis. However, during the following approximately 50-year period, there was limited research, especially from 1948 to 1970, when fewer than 10 publications were published, which were weakly interrelated. This phase involved research related to drug treatment and the analysis of blood chemical components. Due to the lack of in-depth research, coherent clusters were not formed. After 1990, the research focused on management in developed countries and in collecting demographic data. This demonstrates that, initially, developing countries had limited knowledge on CKD and lacked disease management experience. Furthermore, epidemiological surveys of patient groups and related factor analyses became research trends during the early management of CKD, laying the foundation for the next phase of research on the clinical complications and their management.

Phase 2 was a steady growth stage (1999–2010). Significant progress was made in the research on CKD management at this stage. The management of complications, such as anemia, cardiovascular diseases, and dialysis-related calcium–phosphorus metabolism disorders, were clearly clustered. As shown in Figure 3A, blood pressure, obesity, proteinuria, and diabetes were found to be closely related to the progression of CKD. Furthermore, concurrent cardiovascular disease and infections were identified as risk factors for increased CKD mortality rates. In 2006, World Kidney Day promoted the slogan “Care for Health, Protect the Kidney—Early Diagnosis, Active Prevention.” Researchers began to focus on managing hypertension and the early screening of various indicators.^[17–19]^ CKD detection aids in implementing early intervention measures to reduce the risk of cardiovascular events, renal failure, and death associated with CKD. Various research findings^[20–23]^ have shown that early interventions and management can effectively slow CKD progression, improve quality of life, and reduce medical costs. During this stage, “quality of life” emerged as a keyword for the first time, indicating that the concept of chronic disease management had shifted from “duration of life” to “quality of life.” Exploratory studies provided evidence-based support for improving management models and guidelines during the subsequent stages.

Phase 3 was a rapid development phase (2011–2022). During this period, there was a rapid increase in CKD management research. The World Health Organization released declarations concerning kidney disease-related hypertension, diabetes, and cardiovascular disease between 2009 and 2011. After 2011, a vast amount of research on this topic emerged, including the studies by Tuttle et al, which had a significant impact.^[24,25]^ Therefore, numerous guidelines and consensuses were established, indicating that CKD management had entered the standardized management phase.^[10,26]^ The cluster analysis shown in Figure 4 demonstrates that, currently, researchers not only emphasize the management of complications and risk factors, but also focus on patient health management methods; however, initially, researchers explored the quality of life of patients during the previous period. During this phase, topics focused on patient self-management, case management, telemedicine, and primary healthcare formed new clusters.^[27,28]^ With improvements in public education and disease awareness, a better quality of life requirement was proposed, and methods for managing chronic diseases were particularly important during this phase. The traditional medical model, which focuses on disease treatment, has become outdated; the new model for chronic disease management emphasizes a patient’s primary role in disease prevention and control. Recently, through exploration and practice, patient self-management and case management models have gradually demonstrated the advantages of chronic disease management. Furthermore, a combination of telehealth services and health management models is emerging in CKD management, achieving significant results. Havas et al proposed a CKD self-management support program and developed a CKD system for participating patients.^[29]^ Patients can customize support programs based on their condition to meet their nutritional and exercise needs, thereby improving their quality of life, and slowing disease progression. CKD management systems are not limited to data storage; they also provide early disease screening, data organization, data analysis, health consultation, and guidance services. Therefore, this system has become an essential part of CKD management.^[30]^ As shown in Figure 5A, these topics became research hotspots between 2016 and 2018. With the advent of the big data era, the Internet and information technology applications have become new trends in disease management. After 2019, research mainly focused on managing critically ill patients with COVID-19 and kidney disease, suggesting that during CKD management, suggesting that not only should the impact of preexisting conditions on progression be managed during CKD management, but that rapid deterioration due to external infections should also be considered. Healthcare professionals should consider making graded management plans for patients with large-scale infections to improve the predictive and emergency management capabilities of the healthcare system further.

Few studies have investigated the course of chronic renal failure management, with even fewer bibliometric analyses on the dynamic evolution of CKD management. Thus, this study can serve as a reference for other researchers.

### 4.1. Limitations

This study had some limitations. It only analyzed the Web of Science Core Collection database and included only studies published in English. Therefore, literature from non-native English-speaking countries might have been under-represented, which may have biased the research outcomes. Additionally, the reliability of the study requires validation using additional data sources.

## 5. Conclusions

With the continuous exploration and research on CKD management in various countries, the management of complications, such as hypertension, heart disease, and diabetes, has constantly improved. Furthermore, management methods are gradually becoming standardized and the quality of life of patients has noticeably improved. The diversification of management forms has gradually gained the attention of clinical experts. Therefore, we speculate that, during the next phase, establishing management models and applying management tools will remain the main research hotspots in the management of CKD. Furthermore, there is likely to be a more concentrated focus on individualized and detailed management models. Moreover, with the popularization of smartphones and the application of big data, the “Internet Plus” and smart management systems will become mainstream tools for CKD management.

## Acknowledgments

This work was supported by the National Natural Science Foundation of China (grant number: 2019MSXM088) (grant number: 2022yc-jckx20012). We thank Lian Tan for her valuable contributions to this research and Prof Ding Fu for her useful suggestions and guidance.

## Author contributions

**Conceptualization:** Li Dong.

**Data curation:** Li Dong.

**Formal analysis:** Li Dong.

**Funding acquisition:** Li Dong.

**Project administration:** Li Dong.

**Writing – original draft:** Li Dong.

**Writing – review & editing:** Li Dong, Lian Tan.
